# O017. Cortical functional correlates of responsiveness to short-lasting preventive intervention with ketogenic diet (KD) in migraine: a multimodal evoked potentials study

**DOI:** 10.1186/1129-2377-16-S1-A58

**Published:** 2015-09-28

**Authors:** Martina Bracaglia, Gianluca Coppola, Cherubino Di Lorenzo, Davide Di Lenola, Francesco Pierelli

**Affiliations:** 1 Department of Medico-surgical Sciences and Biotechnologies, Sapienza University of Rome Polo Pontino, Latina, Italy; 2 Department of Neurophysiology of Vision and Neuro-ophthalmology, G. B. Bietti Foundation-IRCCS, Rome, Italy

## Background

Ketogenic diet (KD) - a dietetic regimen that mimics fasting in producing ketone bodies - seems to have a role in preventing migraine. The molecular mechanisms underpinning ketogenic diet effectiveness are only partially clear.

## Aim

With the aim of identifying cortical electrofunctional correlates of responsiveness to short-lasting preventive intervention with KD in migraine, we recorded visual (VEPs) and somatosensory (SSEPs) evoked-potentials before and after 1-month intervention with KD.

## Methods

Sixteen interictal migraine without aura patients (MO, ICHD-II code 1.1) underwent VEPs (right eye stimulation, 600 sweeps, 3.1Hz reversal rate, 15 min of arc check) and median nerve SSEPs (right stimulation, 500 sweeps, 4.4 repetition rate, 1.2 motor threshold) recordings, before and during ketogenesis, confirmed by urinary sticks. We measured VEPs N75-P100 and SSEPs N20-P25 amplitudes respectively in 6 and in 2 sequential blocks of 100 sweeps, and habituation as the slope of the linear regression between block 1 to 2 for SSEPs or between 1 to 6 for VEPs.

## Results

After 1-month of KD, a significant reduction of mean migraine frequency (from a mean of 4.1 to 1.4 attacks/month, p < 0.001) and duration (from 51.9 to 16.3 hours/month, p < 0.001) was observed. KD did not change 1^st^ SSEP and VEP block of 100 sweeps, but significantly induced normalization of the interictally reduced VEPs (from +0.09 to -0.14, p = 0.017) and SSEPs (from 0.38 to -0.48, p = 0.002) habituation during the subsequent blocks.

## Conclusions

We found evidence for KD-induced changes at cortical level in parallel to an improvement of migraine. Since KD was able to restore normal EPs habituation curves during stimulus repetition without changing significantly early amplitude responses, we hypothesize that KD acts on habituation via an enhancement of late GABA inhibition.

Written informed consent to publication was obtained from the patient(s).

